# *Piper nigrum* extract suppresses tumor growth and enhances the antitumor immune response in murine models of breast cancer and melanoma

**DOI:** 10.1007/s00262-023-03487-3

**Published:** 2023-07-18

**Authors:** Paola Lasso, Laura Rojas, Cindy Arévalo, Claudia Urueña, Natalia Murillo, Paula Nossa, Tito Sandoval, Luis Carlos Chitiva, Alfonso Barreto, Geison M. Costa, Susana Fiorentino

**Affiliations:** 1 Grupo de Inmunobiología y Biología Celular, Pontificia Universidad Javeriana, Carrera 7a. No. 43-82, Ed. 50, Lab. 101, Bogotá, C.P. 110211 Colombia; 2 Grupo de Investigación en Fitoquímica, Pontificia Universidad Javeriana, Bogotá, Colombia

**Keywords:** Breast cancer, Melanoma, *Piper nigrum*, Immunomodulation, Antitumor, Plant extracts

## Abstract

Although the antitumor effect of *P. nigrum* has been widely studied, research related to its possible immunomodulatory effects is relatively scarce. Here, the antitumor and immunomodulatory activity of an ethanolic extract of *P. nigrum* were evaluated in the murine models of 4T1 breast cancer and B16-F10 melanoma. In vitro evaluations showed that the *P. nigrum* extract has cytotoxic activity, induces apoptotic cell death, and has a pro-oxidant effect in both cell lines, but it regulates glucose uptake differently in both lines, decreasing it in 4T1 but not in B16-F10. *P. nigrum* extract significantly reduced tumor size in both models and decreased the occurrence of macrometastases in 4T1 model. Evaluation of immune subpopulations by flow cytometry revealed that the *P. nigrum* extract significantly increases the frequency of dendritic cells and activated CD8^+^ T cells and decreases the frequency of myeloid-derived suppressor like cells and Tregs in the tumor microenvironment of both models but with different dynamics. Our findings strongly suggest that the *P. nigrum* extract exerts immunomodulatory functions, slightly related to the modulation of cellular energy metabolism, which could ultimately contribute to the promising antitumor effect of *P. nigrum*.

**Supplementary Information:**

The online version contains supplementary material available at 10.1007/s00262-023-03487-3.

## Introduction

*Piper* species, widely distributed in tropical and subtropical regions of the world, are used in the Ayurvedic system of medicine in India as well as in folk medicine in Latin America and the West Indies. Some species, like *P. aduncum* and *P. hispidum* are used for stomach pain and insect repellants. *P. amalago* distributed from Mexico to Brazil is used to relieve chest pain and as an anti-inflammatory agent [1, 2]. The roots and fruits of *P. chaba* and *P. futokadsura* are useful in asthma, bronchitis, fever, and hemorrhoidal conditions [3].

*P. nigrum* is widely used in folk medicine and has been used for diarrhea, indigestion, and respiratory system problems [4]. Surprisingly, *P. nigrum* has also shown significant in vitro and in vivo anticancer activity in many cancer models, including breast, prostate, colon, rectal, lung, melanoma, ovarian, and cervical cancers, among others [5]. The major compounds found in *P. nigrum* extract are alkaloids, including piperine, chavicine, piperidine, pellitorine, and piperedine, while flavones, flavanones, terpenes, steroids, lignans, neolignans, and alkamides are other main compounds [6, 7]. Interest in piperines and their biological activity is not recent, these compounds have been characterized for their anti-inflammatory, immunomodulatory, and anticancer effects [5].

The ethanolic extract as well as the one obtained by supercritical fluids extraction, rich in piperines, present interesting cytostatic activity by inducing arrest in the G1 or G2/M phases of the cell cycle, respectively, in addition to differential cytotoxicity depending on the cell line, apparently due to a p53-dependent effect. Interestingly, the complete extract is more cytotoxic than the two main alkaloids, piperine and pellitorine, and has better specificity for tumor cells than the isolated compounds [8]. A recent clinical study in patients with osteoarthritis showed that the administration of turmeric extract, ginger, and black pepper significantly decreased serum PGE2 levels, in the same way as naproxen used in the control group [9], which may represent an interesting solution for patients allergic to nonsteroidal anti-inflammatory drugs. Some clinical trials are ongoing, studying its anti-inflammatory or adjuvant bioavailability activity, which has been widely reported.

A recent review summarizes a large number of biological activities studied for piperines in cancer [5] and the immune response [10]. In fact, piperine extracts exert an antimetastatic effect, inhibiting the secretion of metalloproteases in various tumor cells as well as the epithelial-mesenchymal transition and angiogenesis. Despite a large number of studies, little is known about its activity on the antitumor immune response and even less about its effect on different immunosuppressive cells. In this work, the antitumor and immunomodulatory activities of an ethanolic extract of *P. nigrum* were evaluated in the metastatic 4T1 breast cancer and B16-F10 melanoma murine models. We found a significant reduction in tumor size in both models and a decrease in macrometastasis in the 4T1 model, along with an increase in the frequency of dendritic cells and activated CD8^+^ T cells. A reduced frequency of myeloid-derived suppressor-like cells (MDSC-LC) and Tregs in the tumor microenvironment was also evidenced. These results suggest that the modulation of the suppressive immune response could underlie the promising antitumor effect of *P. nigrum* extract.

## Materials and methods

### Plant material

Fresh fruits of *Piper nigrum* were collected in Putumayo, Colombia (0°37′45′′ N, 76°51′55′′ W) and identified by Néstor García in the herbarium of the Pontificia Universidad Javeriana (voucher specimen number 30548). The plant material (80 g) was extracted as previously described [11]. Briefly, the dried and ground fruits were extracted by percolation with EtOH 96% and concentrated under reduced pressure by rotary evaporation. Dried extract was stored at room temperature in amber vials duly labeled for later analysis. The P2Et extract, used as positive control for our previous results [12–16], was produced and characterized as previously described [17, 18]. For the in vivo experiments, the extracts were resuspended in PBS, and for in vitro experiments they were resuspended in Ethanol.

### Chromatographic analysis by UPLC-PDA and LC–MS-Q-TOF

The UPLC-PDA analysis was performed with an Acquity UPLC H-class (Waters, Milford, MA, USA), equipped with photodiode array detector (PDA), quaternary pump, on-line degasser and autosampler. Chromatographic separation was performed on a Phenomenex® Kinetex EVO C18 column (100 × 2.1 mm, 2.6 µm, 100 Å), at 30 °C. The mobile phase used was water with 0.1% formic acid (A) and acetonitrile (B) at a flow rate of 0.40 mL/min. The elution gradient was performed as follows: 3% B for 0 to 3 min, 3 to 95% B for 3 to 30 min, 95% B for 30 to 32 min, 95 to 3% B for 32 to 35 min and 3% B for 35 to 40 min. The samples were prepared at a concentration of 1 mg/mL in MeOH LC–MS, filtered through syringe filters (PTFE 0.22 μm and 13 mm in diameter, Millipore® Millex) and 2 μL of each sample were injected. The wavelength used was 274 nm, with spectra acquired over a range of 200–450 nm. For LCMS analysis, same chromatographic conditions were employed. A Nexera X2 LCMS Q-TOF 9030 Shimadzu® chromatograph (Shimadzu®, Duisburg, Germany) with electrospray ionization was used. MS data was acquired in positive mode in an *m/z* range from 100 to 1800, using a full scan (Full-MS) and MS/MS. After the acquisition of the LCMS chromatograms, the raw data were exported in.mzXML format and processed in the free software MZmine version 2.53 (http://mzmine.github.io/) where a deconvolution, alignment and integration process were performed. For the annotation of the characteristics found, mass accuracy (maximum mass error 10 ppm), isotopic pattern distribution, adduct formation and elution order of the compounds based on chromatographic conditions were considered and different online public databases such as METLIN (http://metlin.scripps.edu), KEGG (http://genome.jp/kegg), HMDB (https://hmdb.ca/), PubChem (https://pubchem.ncbi.nlm.nih.gov/), ChEBI (https://www.ebi.ac.uk/chebi/) y NP Atlas (https://www.npatlas.org/). Compounds from primary metabolism were discarded in this process.

### In vitro cytotoxicity assays

MTT (3-(4,5-dimethylthiazol-2-yl)-2,5-diphenyltetrazolium) assay was used to evaluated in vitro cytotoxicity of *P. nigrum* extract as previously reported [13]. The IC_50_ value (50% inhibition of cell growth) was calculated using GraphPad Prism version 8.1.1 for Mac OS X statistics software (GraphPad Software, San Diego, CA).

### Proliferation assay

B16-F10 and 4T1 cells were seeded in 12-well plates at a density of 26,000 cells/cm^2^ and treated with the IC_50_ or IC_50_/5 of P2Et or *P. nigrum* extract. After 6, 12, and 24 h, cells were collected and counted with 0.4% trypan blue. Using the exponential growth method (Mathusian), the population doubling time (PDT) was calculated through GraphPad Prism version 8.1.1 for Mac OS X statistics software (GraphPad Software, San Diego, CA).

### Annexin V and PI double-staining assay

Phosphatidylserine (PS) externalization was assessed by flow cytometry using Annexin V (Molecular Probes, Carlsbad, CA, USA) and propidium iodide (PI) (Sigma, Saint Louis, MO, USA), as previously reported [18, 19]. Briefly, 2 × 10^5^ cells were treated with the IC_50_ and IC_50_/2 of the *P. nigrum* extract (resuspended in ethanol) and IC_50_ of doxorubicin (resuspended in DMSO) at 0.2374 µM for 4T1 or 0.022 µM for B16-F10 for 24 h. Flow cytometry analysis was performed on a FACSAria II-U (BD Biosciences, Washington, WA) and the data was analyzed using FlowJo v10.8.1 software (BD Life Sciences). The assays were performed in triplicate.

### ROS measurement

The production of ROS was evaluated using the probe 2′,7′-dichlorodihydrofluorescein diacetate (H_2_DCFDA) (Sigma Aldrich, Saint Louis MO, USA) as previously described [19]. Briefly, 2 × 10^5^ cells were plated and treated with the IC_50_ and IC_50_/5 of the *P. nigrum* extract, IC_50_ and IC_50_/5 of the P2Et extract (antioxidant control), IC_50_ of doxorubicin (positive control, 0.2374 µM for 4T1 and 0.022 µM for B16-F10), DMSO (doxorubicin vehicle; 0.02%) or ethanol (extract vehicle, 0.02%) for 6, 12 and 24 h were used. Samples were acquired using an FACSAria II-U (BD Biosciences) and analyzed with FlowJo v10.8.1 software (BD Life Sciences). The assays were performed in triplicate.

### Glucose uptake assay

1 × 10^6^ cells were seeded and treated with the IC_50_ and IC_50_/5 of the *P. nigrum* extract, IC_50_ and IC_50_/5 of the P2Et extract (positive control), rotenone (positive control, 1 µM for 4T1 and 50 µM for B16-F10), or ethanol (extract and rotenone vehicle, 0.02%) for 6 and 12 h. After treatments, cells were incubated with 2-NBDG (Invitrogen Molecular Probes) for 30 min at 37 ℃, as previously described [19]. Cells were acquired using a Cytek Aurora Cytometer (Cytek Biosciences, Fremont, CA, USA) and analyzed with FlowJo v10.8.1 software (BD Life Sciences). Experiments were performed in triplicate.

### Measurement of mitochondrial membrane potential

The mitochondrial membrane potential of cells was evaluated using JC-1 dye (Sigma, St. Louis, MO) as previously reported [13]. Briefly, cells were treated with the IC_50_ and IC_50_/5 of the *P. nigrum* extract and the controls for 6 h and 12 h, as previously published [19] (Supplementary Materials and Methods). The cells were acquired on a FACSAria II-U (Becton Dickinson, BD, NJ, USA) and analyzed with FlowJo v10.8.1 software (BD Life Sciences) which calculated the red/green fluorescence ratios.

### Cell culture and reagents

B16-F10 and 4T1 cells were cultured in a 37 °C and 5% CO_2_ incubator with RPMI-1640 (Eurobio, Toulouse, France) with 10% heat-inactivated fetal bovine serum (FBS), 2 mM L-glutamine, 100 U/mL penicillin, 100 μg/mL streptomycin, 0.01 M HEPES buffer, and 1 mM sodium pyruvate [19]. Abs used for cell surface and intracellular staining were previously described [19].

### Mice

Young (6 to 12 weeks old) female C57BL/6NCrl and BALB/cAnNCrl mice were housed at the animal facilities of the Pontificia Universidad Javeriana (PUJ, Bogotá, Colombia) following the established protocols of the Ethics Committee of the Faculty of Sciences, PUJ, and National and International Legislation for Live Animal Experimentation (Colombia Republic, Resolution 08430, 1993; National Academy of Sciences, 2010). Each protocol was approved by the animal experimentation committee of PUJ (FUA-093–20) (Supplementary Materials and Methods).

### Acute toxicity evaluation

BALB/cAnNCrl and C57BL/6NCrl mice were intraperitoneally (IP) inoculated with 2000 mg/kg of *P. nigrum* extract and descaling up to 175 mg/kg. Lethal dose 50% (LD_50_) was calculated with Probit version 14 (Minitab Inc.). The dose of P2Et extract was used as previously reported [14].

### In vivo tumor development experiments and treatment

For melanoma tumor induction, C57BL/6NCrl mice were subcutaneously (s.c.) inoculated in the right flank with 1 × 10^5^ viable B16-F10 cells. For the breast cancer murine model, 1 × 10^4^ viable 4T1 cells were s.c. injected into the right mammary fat pad of BALB/cAnNCrl mice as previously described [19]. Five days after tumor cells implantation, 8 mice per group were treated intraperitoneally (i.p.) with 75 mg/Kg (B16-F10 model) or 18.7 mg/Kg (4T1 model) body weight of P2Et extract (positive control), 13 mg/Kg (B16-F10 model) or 15.67 mg/Kg (4T1 model) body weight of *P. nigrum* extract, or PBS as negative control (extract vehicle) two times per week [19, 20]. The size of the tumors was assessed three times per week with Vernier calipers, and the volume was calculated according to the formula V (mm^3^) = L (major axis) x W^2^ (minor axis)/2 [20]. Mice were euthanized and spleen, tumor-draining lymph nodes (TDLN), and tumor were removed and processed. In addition, in the 4T1 breast cancer model, where metastases are clearly visible, the appearance of these in different organs was evaluated.

### Analysis of immune populations and cytokine production by flow cytometry

It was evaluated as previously reported [19]. Briefly, cells were stained with LIVE/DEAD Fixable Aqua and then were stained with the surface antibodies according to the designed multicolor panels (Supplementary Table 1). To cytokine production, spleen cells were cultured with phorbol 12-myristate 13-acetate (PMA) and ionomycin (P/I) or without a stimulus for 6 h and then stained according to the multicolor panel (Supplementary Materials and Methods). The cells were acquired by flow cytometry using the Cytek Aurora Cytometer (Cytek Biosciences, Fremont, CA, USA), and the results were analyzed using FlowJo v10.8.1 software (BD Life Sciences). For dimensionality reduction analysis, the OMIQ platform (https://www.omiq.ai/) was used. Single live CD45 cells for each file were concatenated for analysis by opt-SNE dimensionality reduction [19]. Multifunctional analyses were performed using a Boolean gating strategy. The data are presented using Pestle v2.0 and SPICE v6.1 software (the National Institutes of Health, Bethesda, MD) [19, 21].

### Statistical analysis

Statistical analysis of the significance between two groups was calculated using the Mann Whitney U test. Differences among subject groups were evaluated using Kruskal Wallis and Dunn’s posttest for multiple comparisons. GraphPad Prism version 8.1.1 for Mac OS X statistics software (GraphPad Software) was used for the statistical analyses.

## Results

### Chromatographic analysis

**Fig. 1:**
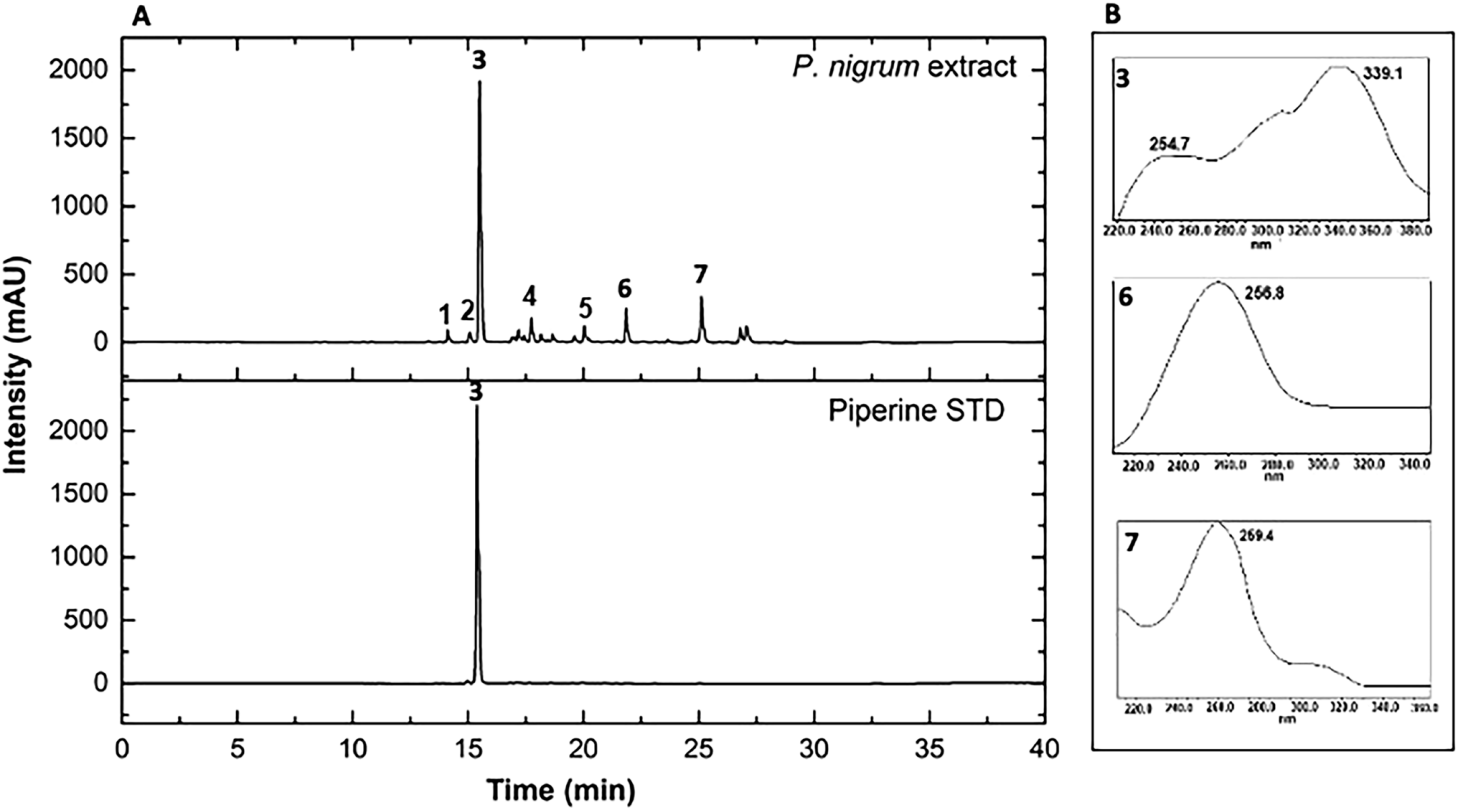
**A** Top: UPLC-DAD chromatogram of the *P. nigrum* extract at 254 nm showing the major peaks at different retention times. Bottom: Comparison of compound 3 with the commercial standard (STD) of piperine. **B** UV spectra of the major peaks identified as (3) piperine, (6) piperolein A, and (7) piperolein B

Chemical analysis by UPLC-PDA of the crude extract of *P. nigrum* at the wavelength of 254 nm showed a diversity of compounds of different polarities, observed across the different retention times. Three major peaks were observed at Rt = 15.81, 22.0, and 25.35 min, identified as piperine (3), piperolein A (6), and piperolein B (7), respectively. Compound 3 was compared with the commercial standard (STD) based on its retention time and UV spectra, unlike compounds 6 and 7, which were tentatively identified (Fig. 1). Additionally, the phytochemical profile of the *P. nigrum* fruit extract was also analyzed by LC–MS-QTOF. The results confirmed the presence of piperine, among other secondary alkaloid-type metabolites corresponding to the alkamides, such as piperettine, trichostachine, piperolein A, and piperolein B (Table 1; Supplementary Fig. 1). These compounds have been previously reported for this species in other investigations [22–24].

**Table 1:** Compounds tentatively identified from *P. nigrum* fruit extract by LC-DA-MS-Q-TOF

Peak number	RT (min)	Compound	UV (nm)	Molecular formula	*m/z* [M + H]^+^	Error (ppm)	MS/MS fragment
1	14.16	Piperettine	**–**	C_19_H_21_NO_3_	312.1246	5	**–**
2	15.18	Trichostachine	**–**	C_16_H_17_NO_3_	272.1283	− 5	**–**
3	15.81	Piperine*	254.7, 339.1	C_17_H_19_NO_3_	286.1392	− 0.7	201, 171, 143, 115
4	17.94	Not identified	**–**	C_19_H_21_NO_3_	311.1521	4	**–**
5	20.07	Not identified	**–**	C_17_H_21_NO_3_	287.1521	3	**–**
6	22.00	Piperolein A	256.8	C_19_H_25_NO_3_	316.1905	− 1	285, 271, 115
7	25.35	Piperolein B	259.4	C_21_H_29_NO_3_	344.2208	2	285, 271, 115

*Confirmed by injection of authentic standard

### In vitro activity of *P. nigrum* extract on tumor cells

**Fig. 2:**
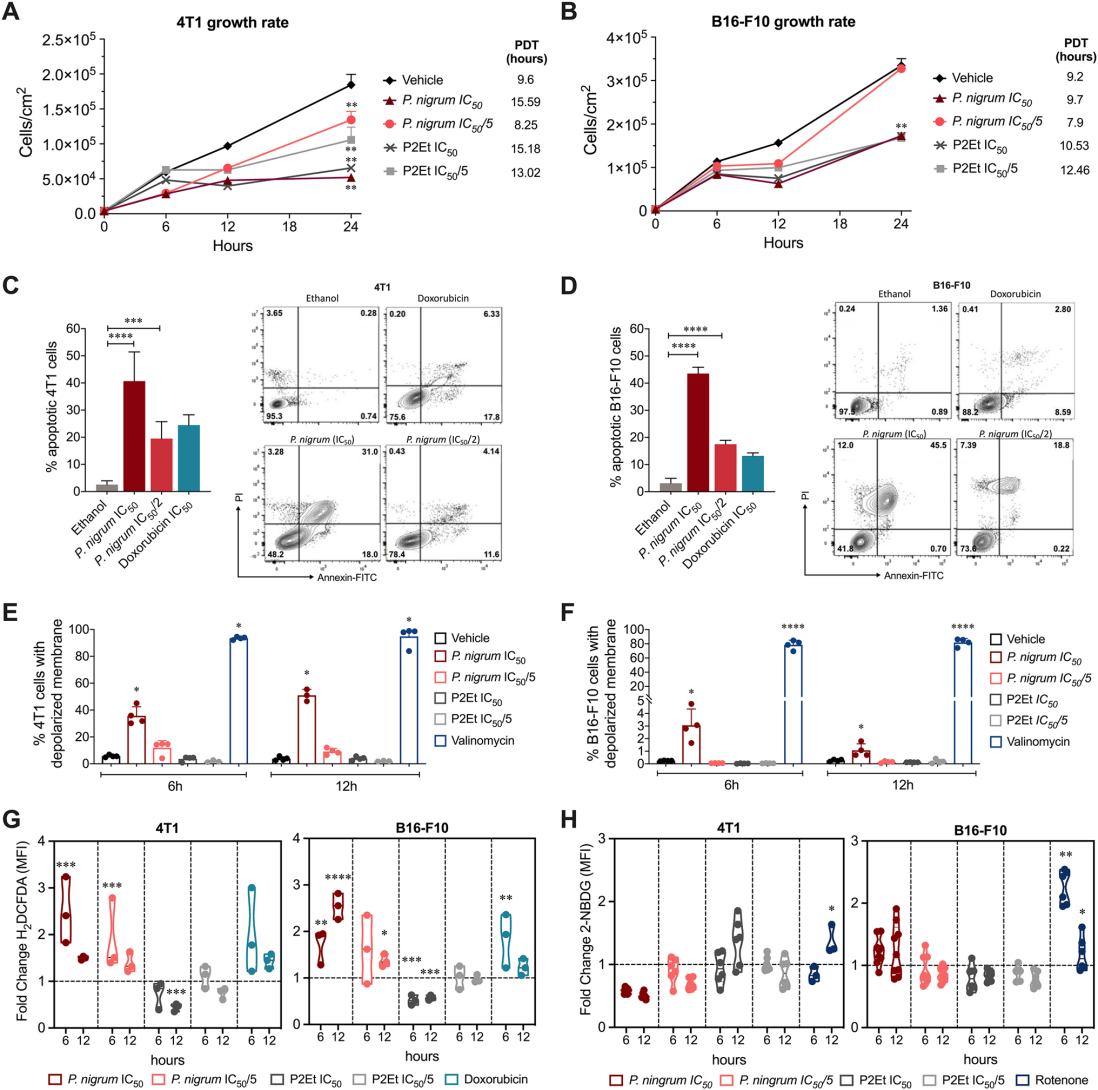
In vitro activity of ethanolic *P. nigrum* extract. Cell count per cm^2^ of 4T1 (**A**) and B16-F10 (**B**) cells after treatments for 0, 6, 12, and 24 h. Population doubling times (PDT) are shown. Representative contour plots of 4T1 (**C**) and B16-F10 (**D**) cells incubated with IC_50_ and IC_50_/2 of *P. nigrum* extract, ethanol (negative control) or IC_50_ of doxorubicin (positive control) for 24 h. Representative flow cytometry analysis showing necrotic (Annexin V^−^, PI^+^), late apoptotic (Annexin V^+^, PI^+^), early apoptotic (Annexin V^+^, PI^−^) and viable (Annexin V^−^, PI^−^) cells. Frequency of 4T1 (**C**) and B16-F10 (**D**) cells in apoptosis (sum of early and late apoptosis) expressed as mean ± SEM for three independent experiments. Frequency of 4T1 (**E**) and B16-F10 (**F**) cells with depolarized membrane evaluated by flow cytometry after treatments for 6 and 12 h. **G.** Fold change of H_2_DCFDA MFI after the treatments with IC_50_ and IC_50_/5 of *P. nigrum* extract, IC_50_ and IC_50_/5 of P2Et extract, or IC_50_ of doxorubicin (positive control) for 6, 12, and 24 h in both cell lines. **H.** Fold change of 2-NBDG MFI after treatments with IC_50_ and IC_50_/5 of *P. nigrum* extract, IC_50_ and IC_50_/5 of P2Et extract, or rotenone (positive control) for 6 and 12 h. In all cases, fold change was determined using MFI of each treatment relative to vehicle (ethanol or DMSO). Data of three independent experiments are shown. ^*^p < 0.05; ^**^p < 0.01; ^**^p < 0.001; ^***^p < *0.0001*

The extract reduced 4T1 and B16-F10 cell viability in a dose-dependent manner with an IC_50_ of 124.3 μg/ml and 27.52 ± 1.23 μg/ml, respectively (Supplementary Fig. 2). In addition, the standardized extract of *P. nigrum* significantly reduced the proliferation of 4T1 (Fig. 2A) and B16-F10 (Fig. 2B) at both IC_50_ and 1/5 of the IC_50_ in the first 12 h. Then, we evaluated the type of tumor cell death induced by *P.* *nigrum* extract, finding that the treatment for 24 h induces apoptosis in both 4T1 (Fig. 2C) and B16-F10 (Fig. 2D) cells. In addition, 4T1 (Fig. 2E) and B16-F10 (Fig. 2F) treated with *P.* *nigrum* extract at different concentrations for 6 and 12 h showed mitochondrial membrane depolarization with the highest dose at both treatment times. According to this result, *P. nigrum* extract also presented pro-oxidant activity in both 4T1 and B16-F10 cells; however, while in 4T1 it decreases over time, in B16-F10 it increases at 12 h with the IC_50_, suggesting a difference in the mechanisms activated by *P.* *nigrum* extract and involved in ROS induction (Fig. 2G). As expected, P2Et extract confirms its intracellular antioxidant activity in both populations, while doxorubicin confirms its pro-oxidant effect, as observed in previous reports (Fig. 2G). Regarding the effect of the extract on glucose uptake in the 4T1 cell line, *P.* *nigrum* induces a dose-dependent decrease in intracellular glucose uptake, in contrast to a small increase in intracellular glucose uptake observed in B16-F10 cells (Fig. 2H). Rotenone increased glucose uptake in both cell lines, although more markedly in B16-F10 (Fig. 2H), suggesting a greater metabolic plasticity of B16-F10 cells and perhaps a greater ability to evade treatment with metabolism-regulating drugs.

### *P. nigrum* extract decreases tumor size and metastases

**Fig. 3:**
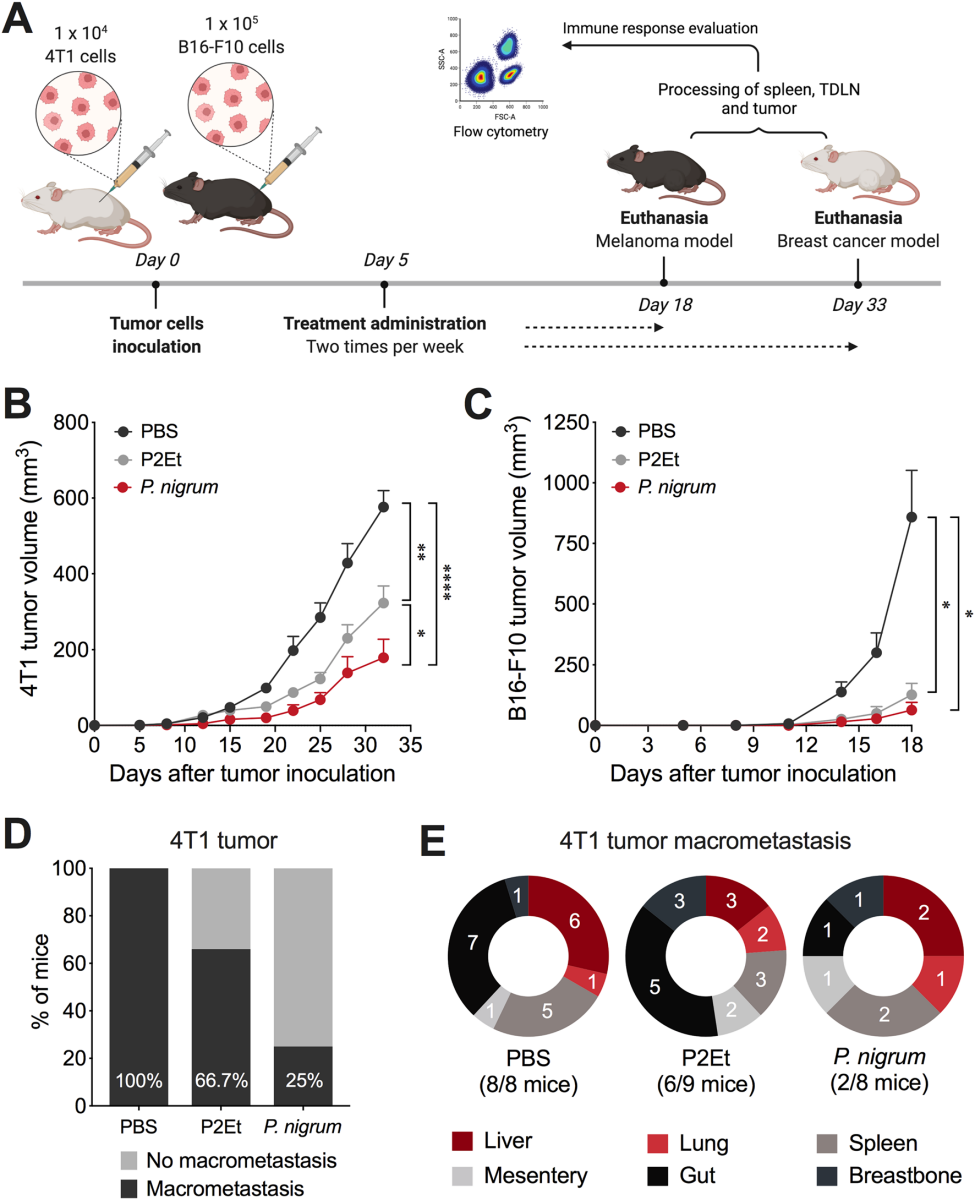
In vivo* P. nigrum* treatment delays tumor growth. **A.** Experimental design to evaluate the effect of *P. nigrum* extract in 4T1 and B16-F10 tumor bearing mice. Tumor was established by injection of 4T1 and B16-F10 cells in young (6 to 12 weeks old) female BALB/cAnNCrl or C57BL/6NCrl mice and 5 days after tumor cell injection, treatments were administrated two times per week until the end of the experiment. Tumor volume in 4T1 (**B**) and B16-F10 (**C**) tumor-bearing mice treated with each treatment.** D** Bars showing the percentage of mice that developed macrometastasis in 4T1 breast cancer model. **E** Distribution of multi-organ metastasis of 4T1 tumors for all groups. The numbers on the pies show the mice with macrometastasis and numbers in parenthesis corresponds to the total of mice with macrometastases. The *p* values were calculated using Kruskal – Wallis and Dunn’s posttest for multiple comparisons. *p < 0.05; **p < 0.01; ****p < 0.0001

To evaluate the effect of the *P. nigrum* extract in vivo, the animals transplanted with the 4T1 or B16-F10 tumor cells were treated from day 5 until day 18 for the B16-F10 model and until day 33 for the 4T1 model (Fig. 3A). The *P.* *nigrum* extract therapeutic dose was determined following LD_50_ estimation. To ensure no toxicity, animals were treated with 13 mg/Kg (B16-F10 model) or 15.67 mg/Kg (4T1 model) body weight of *P.* *nigrum* extract, which corresponds to 4 times lower doses than LD_50_. The *P. nigrum* extract significantly decreased tumor size in the 4T1 model (Fig. 3B, Supplementary Fig. 3), as well as in B16-F10 (Fig. 3C, Supplementary Fig. 3), in the same way as the positive control P2Et, although to a greater extent for 4T1 (Fig. 3B). Interestingly, it was observed that macrometastasis of 4T1 cells were controlled to a greater extent by the *P.* *nigrum* extract than even by the P2Et extract (Fig. 3D). Although the distribution of the macrometastasis in the two animals treated with *P.* *nigrum* was varied, P2Et-treated animals showed macrometastasis principally in the gut (Fig. 3E).

### Treatment with *P. nigrum* decreases the immunosuppressive response and induces an adaptive intratumoral immune response

**Fig. 4:**
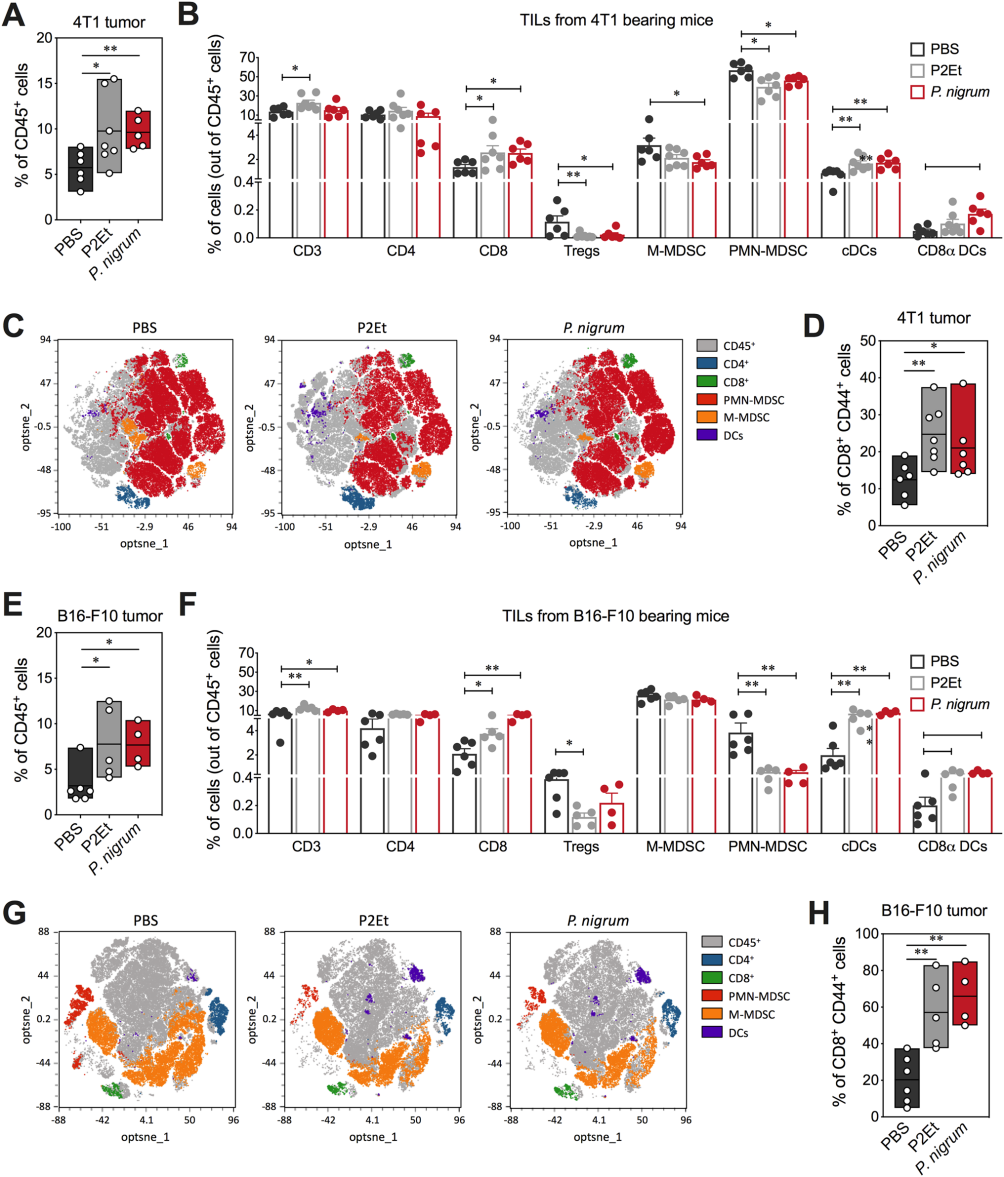
*P. nigrum* extract modulates the tumor microenvironment. **A** Frequency of 4T1 intratumor CD45^+^ cells in mice treated with *P. nigrum*, P2Et (positive control), or PBS (negative control). **B** Overview of the immune cell composition in the 4T1 TME shown in percentage of cells (out of CD45^+^ cells) on a per-mouse basis. **C** opt-SNE visualization of clustering of some subpopulations from 4T1 tumor detected by flow cytometry, each dot corresponds to one single cell. **D** Frequency of activated CD8^+^ T cells (CD44^+^). **E** Frequency of B16-F10 intratumor CD45^+^ cells in mice groups. **F** Overview of the immune cell composition in the B16-F10 TME shown in percentage of cells (out of CD45^+^ cells). **G** opt-SNE visualization of clustering of some immune subpopulations from B16-F10 tumor. **H** Frequency of activated CD8^+^ T cells (CD44^+^). In all cases, data are represented as the mean ± SEM. The *p* values were calculated using Mann–Whitney *U* test. *p < 0.05; **p < 0.01

The fine analysis of the antitumor immune response (Supplementary Fig. 4) induced by the treatment with the *P. nigrum* extract reveals an increase in the infiltrate of CD45 + hematopoietic cells, just as the P2Et extract does, both in 4T1 and in B16-F10 (Fig. 4A, E). In the case of 4T1 tumors, mice treated with *P.* *nigrum* extract presented an infiltrate composed mainly of activated CD8^+^ T cells (CD44 +), conventional dendritic cells (cDCs), and CD8α^+^ DCs, with a significant decrease in Treg, M-MDSC-LC, and PMN-MDSC-LC (Fig. 4B–D). These changes were subtly evident in the opt-SNE analysis (Fig. 4C). In B16-F10 tumors, an increase in activated CD8^+^ T cells (CD44 +), as well as cDCs and CD8α^+^ DCs, and a decrease in PMN-MDSC-LC were also observed, but unlike what was observed in the 4T1 model, the decrease in Tregs was not significant, and a decrease in M-MDSC-LC was not observed (Fig. 4F–H). These results suggest that in both cases, there is an activation of the adaptive immune response, evidenced by the activation of CD8^+^ T cells and the migration of CD8α^+^ DCs to the tumor lumen, which are mainly involved in intratumoral cross-priming [25].

### *P. nigrum* induces a systemic immune response mainly in 4T1

**Fig. 5:**
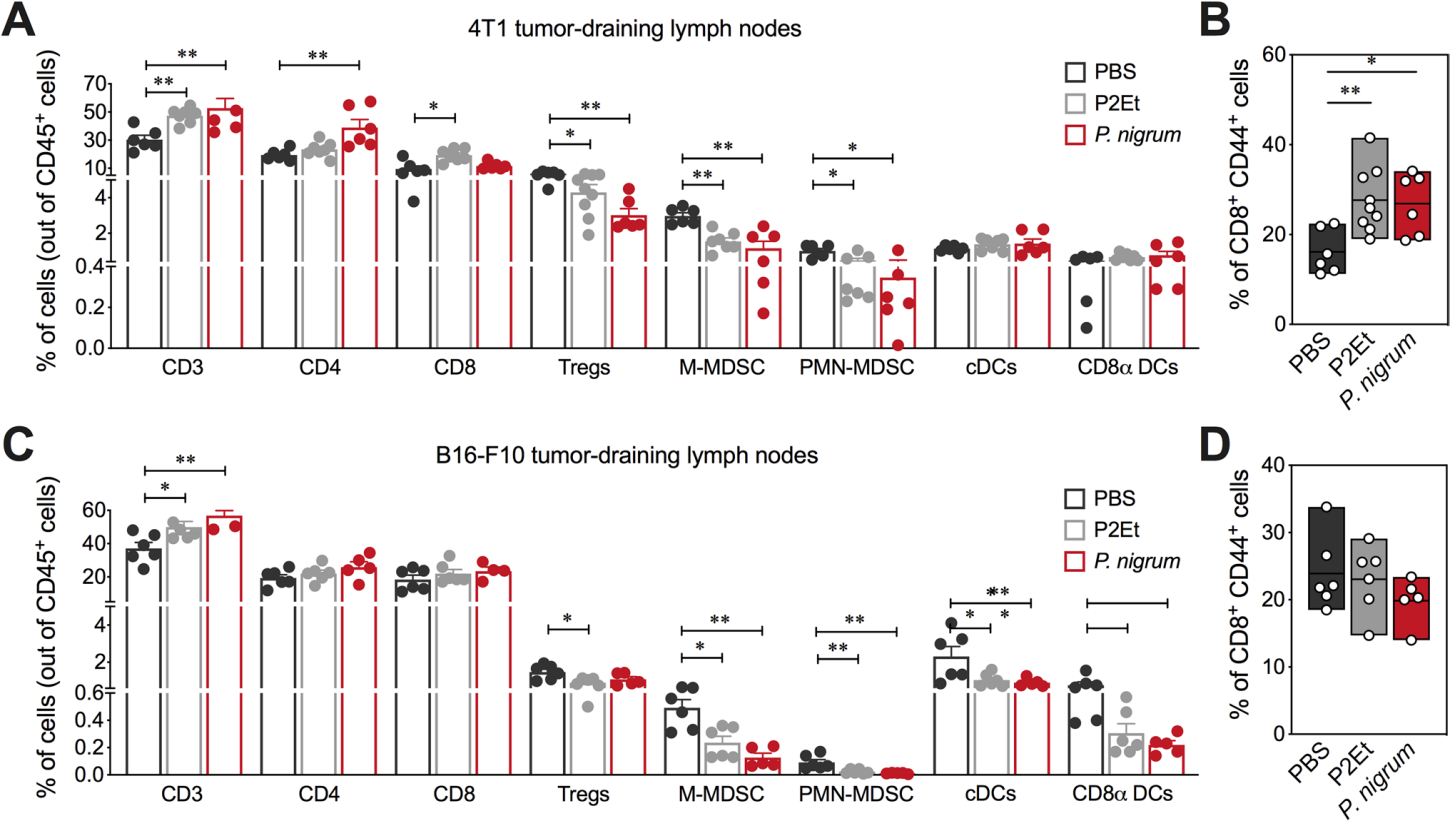
*P. nigrum* extract modulates the immune response in lymph nodes. **A** Overview of the immune cell composition in lymph nodes from 4T1 tumor-bearing mice shown in percentage of cells on a per-mouse basis. **B**. Frequency of activated CD8^+^ T cells (CD44^+^). **C** Overview of the immune cell composition in lymph nodes from B16-F10 tumor-bearing mice shown in percentage of cells on a per-mouse basis. **D** Frequency of activated CD8^+^ T cells (CD44^+^). In all cases, data are represented as the mean ± SEM. The *p* values were calculated using Mann–Whitney *U* test. *p < 0.05; **p < 0.01

Analysis of the infiltrate present in the TDLN of 4T1 transplanted and treated mice, showed that *P.* *nigrum* induced a higher frequency of CD4^+^ T cells and a lower frequency of Treg, M-MDSC-LC and PMN-MDSC-LC (Fig. 5A). In addition, an increase in the frequency of activated CD8 T cells was found (Fig. 5B). In contrast, in the TDLN of animals with B16-F10 tumors, although an increase in CD3^+^ cells was observed in the mice treated with the *P.* *nigrum* extract (Fig. 5C), activated CD8^+^ T cells were decreased (Fig. 5D), suggesting that the immune response induced in these animals is mainly intratumorally, as previously shown (Fig. 4). Add to this, we showed that, as in the 4T1 model, the lymph nodes present a lower frequency of M-MDSC-LC and PMN-MDSC-LC, but in contrast, a decrease in cDCs and CD8α^+^ DCs was observed (Fig. 5C). These results suggest a preference for the induction of an antitumor immune response, preferentially at the tumor site and not in the TDLN in the B16-F10 model.

### *P. nigrum* treatment induces multifunctional T cells

**Fig. 6:**
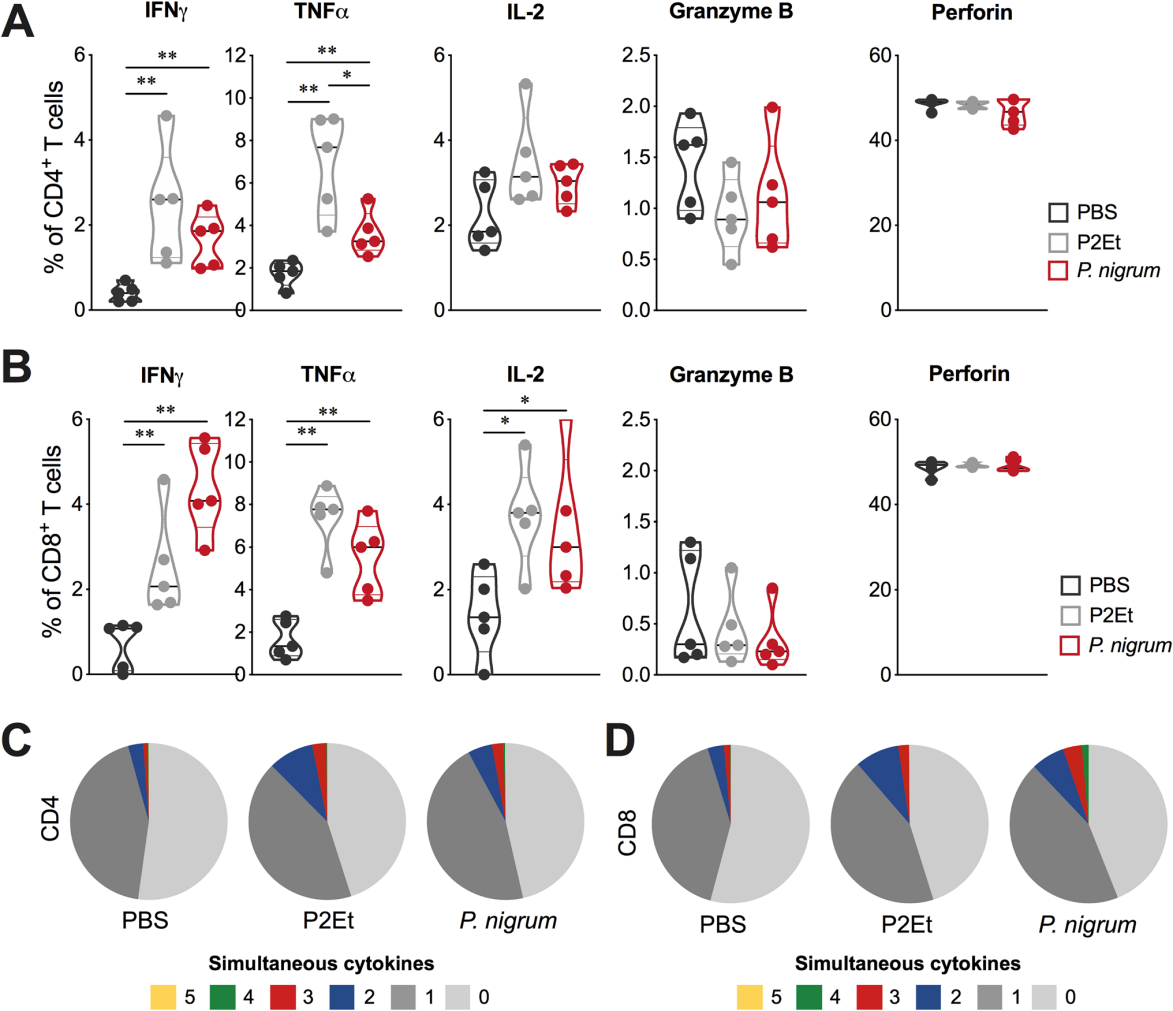
*P. nigrum* extract enhances the functional activity of the T cells in the breast cancer model. Frequency of CD4^+^ (**A**) or CD8^+^ (**B**) T cells from spleen producing IFNγ, TNFα, IL-2, granzyme B and perforin following stimulation with PMA/ionomycin (P/I). Functional activity of CD4^+^ (**C**) or CD8^+^ (**D**) T cells in each mice group determined using a five functions assay to measure simultaneous IFNγ, TNFα, IL-2, granzyme B, and perforin expression after stimulation with P/I. The functional profiles are grouped and color-coded according to the number of simultaneous T cell functions, as shown in the pie charts. Multifunctional analyzes were performed using a Boolean gating strategy with FlowJo v10.8.1 software and subsequently, data were analyzed and plotted with Pestle v2.0 and SPICE v6.1 software. Data are presented by violin plots showing all points with its corresponding median *p < 0.05; **p < 0.01

When evaluated the functionality of T cells in the 4T1 model, a higher frequency of CD4^+^ T cells spontaneously secreting IFNγ or TNFα were generated in response to *P.* *nigrum* treatment compared to control mice, although to a lesser extent than with the P2Et control treatment (Fig. 6A). In contrast, *P nigrum* treatment induced a similar frequency of CD8^+^ T cells secreting IFNγ, TNFα, or IL-2 than P2Et (Fig. 6B). None of the treatments increased the cytotoxicity markers granzyme B and perforin at the time of evaluation, which does not mean that in the face of direct activation with the tumor cell, a tumor-specific cytotoxic activity cannot be evidenced.

**Fig. 7:**
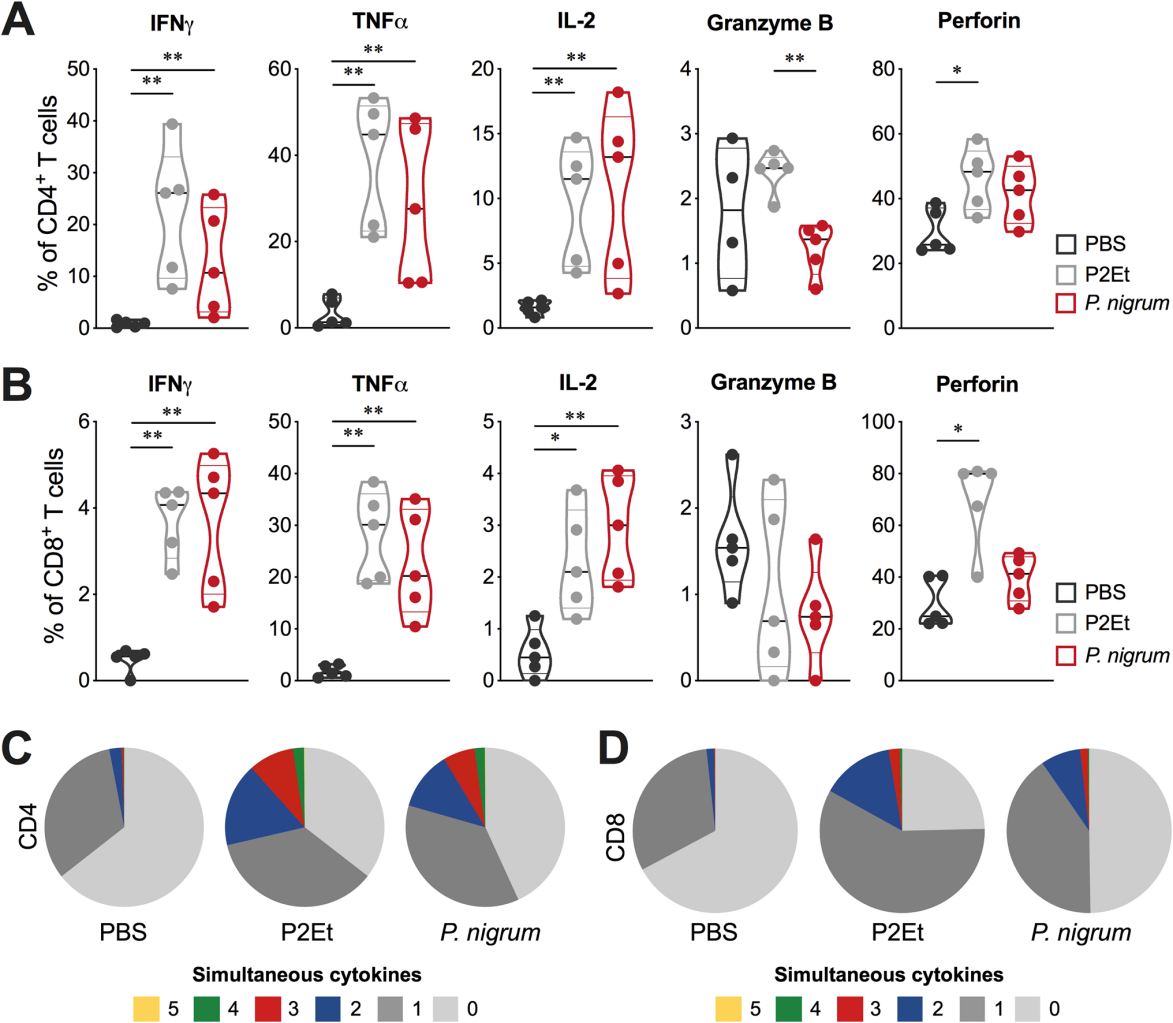
*P. nigrum* extract modulates the functional activity of the T cells in the melanoma model. Frequency of CD4^+^ (**A**) or CD8^+^ (**B**) T cells from spleen producing IFNγ, TNFα, IL-2, granzyme B and perforin following stimulation with PMA/ionomycin (P/I). Functional activity of CD4^+^ (**C**) or CD8^+^ (**D**) T cells in each mice group determined using a five functions assay to measure simultaneous IFNγ, TNFα, IL-2, granzyme B, and perforin expression after stimulation with P/I. The functional profiles are grouped and color-coded according to the number of simultaneous T cell functions, as shown in the pie charts. Multifunctional analyzes were performed using a Boolean gating strategy with FlowJo v10.8.1 software and subsequently, data was analyzed and plotted with Pestle v2.0 and SPICE v6.1 software. Data are presented by violin plots showing all points with its corresponding median *p < 0.05; **p < 0.01

Previous reports have suggested that the quality of the T cell response should be assessed by its polyfunctional activity, known as the ability to produce two or more cytokines simultaneously [26]. Therefore, we extended the assessment of the functional activities of T cells from each mice group stimulated with P/I by simultaneously measuring the production of IFNγ, TNFα, IL-2, perforin, and granzyme B. In summary, *P.* *nigrum* induced a higher frequency of multifunctional CD4^+^ and CD8^+^ T cells, compared to the control group, like that observed with mice treated with P2Et extract (Figs. 6C and 6D). In the B16-F10 model, treatment with *P. nigrum* induced the generation of CD4^+^ T cells that produce IFNγ, TNFα, or IL-2, although to a lesser extent than P2Et extract. However, while in the 4T1 model the range of activated CD4^+^ T cells was from 2 to 12% for both treatments (Fig. 6A), in the B16-F10 model it was from 10 to 60% (Fig. 7A). This important activation was also observed for CD8^+^ T cells, where the frequency of cells producing INFγ and IL-2 was similar but the ability to produce TNFα was considerably higher (Fig. 7B). In CD8^+^ T cells, P2Et extract was capable of inducing an increase in intracellular perforin in both CD4^+^ and CD8^+^ T cells, unlike *P. nigrum* treatment, which does not induce changes. When multifunctionality was evaluated, an increase in the frequency of multifunctional T cells was found in animals treated with *P.* *nigrum*, like that observed in animals treated with P2Et compared to the control group (Fig. 7C, D).

## Discussion

Cancer is a complex disease that involves the uncontrolled development of a tumor cell that has lost its effective communication with the microenvironment [27, 28]. Even though it is assumed today that there are multiple factors involved in the development and progression of tumors, the search for new therapies is still limited. In fact, it has focused on the isolation of molecules that come mostly from natural products, and although they present a defined activity against different molecular targets, they do not allow total control of the tumor [29]. More recently, research has focused on the activation of the antitumor immune response, which has led to the use of combined therapies in which, on the one hand, tumor elimination is allowed but the immunosuppression in the microenvironment is also reduced [30, 31]. Plant-derived polymolecular drugs, better known as herbal drugs, begin to show the multiple facets of their antitumor activity, acting not only on the tumor but also on the immune response [32], as we observed in this work.

A large number of publications have reported the antitumor activity of the extract and its metabolites, as well as the identification of some molecular targets and even the induction of immunogenic cell death through the induction of reticulum stress, which suggests the subsequent activation of the immune response [33], confirmed by our findings. Crude extracts of *P. nigrum* have exhibited multiple bioactivities, including anti-inflammatory, antitumor, and selective killing of tumor cells [34–36]. Moreover, some studies have demonstrated that the main constituents of the *P. nigrum* extract piperine and piperyline, also called trichostachine, possess antitumor properties in vitro and in vivo [37–40], and furthermore, piperyline induces apoptosis and inhibits the differentiation of the osteoblast [41].

The antitumor activity of piperine, one of the main constituents of *P. nigrum*, has been widely described [5, 10, 42], as well as their ability to induce apoptosis, increase ROS, cell cycle arrest, inhibit cancer stem cell renewal, induce reticulum stress, and immunogenic cell death [5, 43, 44]. In vivo, treatment with piperine has shown its antitumor activity in the B16-F10 model, decreasing tumor metastases in the lung and significantly increasing animal survival*.* In addition, in the 4T1 model, piperines have also been found to decrease tumor growth as well as metastasis [5]. However, the relationship between its antitumor activity and its ability to regulate tumor metabolism has not been clearly established, although the role of natural products as regulators of tumor metabolism has been extensively studied. In fact, piperine has similar effects to metformin on glucose metabolism and improves insulin sensitivity while decreases inflammation in obese MSG mice [45, 46]. Edible and medicinal plants have immune modulatory effects as well as metabolic regulation, introducing immunometabolism as a focus in understanding the antitumor activity of plants and other complex products of natural origin [47].

Metabolic reprogramming as a prerequisite for tumorigenicity and immune evasion has been widely documented by Hannahan and Weinberg [28, 48]. Even hyperglycemia and glucose intolerance are negative predictive factors for the appearance of cancer [49]. Recently, glucose metabolism has been directly linked as a target to improve immunotherapies [50]. A negative regulation of the antitumor immune response related to the reduction of NK activity and the increase of MDSC has been related to the presence of lactate in the tumor microenvironment [51]. In fact, MDSCs are increased in the primary tumor of 4T1 vs. B16-F10. Therefore, it is possible that the regulation of extracellular acidity in 4T1 explains the greater anti-metastatic activity observed for this tumor in our work. In addition, a low extracellular pH is related to an anergic state of the T cells, and we found a greater intratumorally CD8 infiltrate, which could be explained by a recovery of the tumor microenvironment. In support of this, it has just been shown that an extract of *Piper nigrum* increases antitumor immunity by regulating the Th1/Th2/Treg ratio [52].

## Supplementary Information

Below is the link to the electronic supplementary material.

Supplementary file1 (PDF 2818 kb)
