# Supplementary material for: Piper nigrum extract suppresses tumor growth and enhances the antitumor immune response in murine models of breast cancer and melanoma
Source: Cancer Immunol Immunother. 2023 Jul 18;72(10):3279–92. doi: 10.1007/s00262-023-03487-3 (PMC10491708; doi:10.1007/s00262-023-03487-3)
Supplement: Supplementary file 1 — Supplementary file1 (PDF 2818 kb) [file 262_2023_3487_MOESM1_ESM.pdf]

SUPPLEMENTARY MATERIALS AND METHODS

Measurement of mitochondrial membrane potential

The mitochondrial membrane potential of 4T1 and B16-F10 cells was detected by flow cytometry, using JC-1 dye (Sigma, St. Louis, MO). Briefly, 1 x 10^5 cells were treated with the IC_{50} and $IC_{50/5}$ of the *P. nigrum* extract, IC_{50} (34.1 µg/ml for 4T1 and 36.4 µg/ml for B16-F10) and $IC_{50/5}$ of the P2Et extract (positive control), valinomycin (positive control, 1 µg/ml), DMSO or ethanol (negative controls, 0.02%) for 6 and 12 h. JC-1 staining solution (2.5 µg/ml in PBS) was added and incubated for 10 min at 37 °C. The cells were acquired on a FACSAria II-U (Becton Dickinson, BD, NJ, USA) and analyzed with FlowJo v10.8.1 software (BD Life Sciences). Experiments were performed in triplicate and the results were expressed as mean ± SEM.

Mice

Mice were maintained in polyethylene cages with food and water provided ad libitum, on a 12-h light/dark cycle at 20–22 ºC and 40–60% humidity.

Abs

The following Abs were used for cell surface staining: anti-CD3 Pacific Blue (clone 17A2), anti-CD8 PE Dazzle 594 (clone 53.6.7), anti-CD45 PE-Cy5 (clone 30-F11), anti-Ly-6G PE-Cy7 (clone 1A8), anti-Ly-6C APC-Cy7 (clone AL-21), anti-PD-L1 PE (clone 10F.9G2), anti-PD-1 APC (clone 29F-1A12), CD11b Alexa Fluor 700 (clone M1/70), anti-CD4 Brilliant Violet 570 (clone RM4-5), anti-CD44 PE-Cy7 (clone IM7), CD25 APC (clone 3C7) (Biolegend, San Diego, CA, USA) and CD11c FITC (clone HL3) (BD Biosciences, San José, CA, USA). The abs for intracellular staining included anti-FoxP3 Alexa Fluor 488 (clone MF23) (BD Biosciences) and anti-CTLA-4 PE (clone UC10-4F10-11) (Biolegend). A LIVE/DEAD Fixable Aqua Dead Cell Stain Kit (Life Technologies, Thermo Scientific, Eugene, OR, USA) was used for dead cell exclusion. The abs used for intracellular cytokines evaluation were anti-IFNγ Alexa Fluor 700 (clone XMG1.2), TNFα PE-Cy7 (clone MP6-XT22), IL-2 FITC (clone JES6-5H4) (BD Biosciences), anti-perforin APC (clone S16009A) and anti-granzyme B PE (QA16A02) (Biolegend) **(Supplementary Table 1)**.

Evaluation of the cytokine production by flow cytometry

Spleen cells were cultured with phorbol 12-myristate 13-acetate (PMA) and ionomycin (P/I) or without a stimulus for 6 hours. The last 5 hours of culture were performed in the presence of brefeldin A (1 µg/ml) (BD Pharmingen). Briefly, 1 × 10^6 cells were stained with LIVE/DEAD Fixable Aqua for 20 minutes in the dark at room temperature. After washing with PBS containing 2% FBS, the cells were stained for 30 minutes at 4 °C in the dark with anti-CD45, anti-CD3, anti-CD4, and anti-CD8 antibodies. Later, the cells were washed, fixed, and permeabilized for final staining with anti-IFNγ, anti-TNFα, anti-IL-2, anti-perforin and anti-granzyme B. Cells were acquired through flow cytometry using the Cytek Aurora Cytometer (Cytek Biosciences), and the results were analyzed using FlowJo v10.8.1 software (BD Life Sciences). Multifunctional analyses were performed using a Boolean gating strategy. The data are presented using Pestle v2.0 and SPICE v6.1 software (the National Institutes of Health, Bethesda, MD) [1].

1. Roederer M, Nozzi JL, Nason MC (2011) SPICE: exploration and analysis of post-cytometric complex multivariate datasets. Cytometry A. 79: 167-74. doi: 10.1002/cyto.a.21015

SUPPLEMENTARY TABLES

Supplementary Table 1. List of antibodies for flow cytometry

Target	Conjugate	Clone	Company	Multicolor panel
CD45	PE-Cy5	30-F11	Biolegend	1
CD3	Pacific Blue	17A2	Biolegend	1
CD4	Brilliant Violet 570	RM4-5	Biolegend	1
CD8	PE-Dazzle 594	53.6.7	Biolegend	1
CD11c	FITC	HL3	BD Biosciencies	1
CD11b	Alexa Fluor 700	M1/70	Biolegend	1
Ly6C	APC-Cy7	AL-21	Biolegend	1
Ly6G	PE-Cy7	1A8	Biolegend	1
CD45	PE-Cy5	30-F11	Biolegend	2
CD3	Pacific Blue	17A2	Biolegend	2
CD4	Brilliant Violet 570	RM4-5	Biolegend	2
CD8	PE-Dazzle 594	53.6.7	Biolegend	2
CD44	PE-Cy7	IM7	Biolegend	2
CD25	APC	3C7	Biolegend	2
CTLA-4	PE	UC10-4F10-11	Biolegend	2
FoxP3	FICT	MF23	BD Biosciencies	2
CD45	PE-Cy5	30-F11	Biolegend	3
CD3	Pacific Blue	17A2	Biolegend	3
CD4	Brilliant Violet 570	RM4-5	Biolegend	3
CD8	PE-Dazzle 594	53.6.7	Biolegend	3
IFNγ	Alexa Fluor 700	XMG1.2	BD Biosciencies	3
TNFα	PE-Cy7	MP6-XT22	BD Biosciencies	3
IL-2	FITC	JES6-5H4	BD Biosciencies	3
Perforin	APC	S16009A	Biolegend	3
Granzyme B	PE	QA16A02	Biolegend	3

SUPPLEMENTARY FIGURES

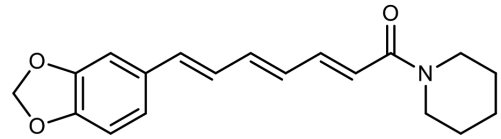

1

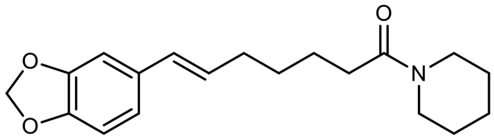

4

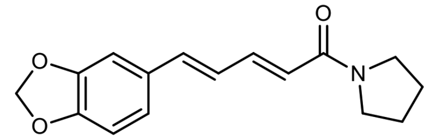

2

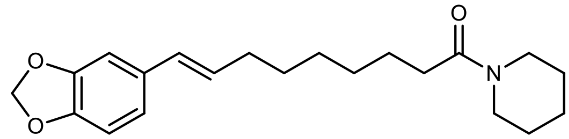

5

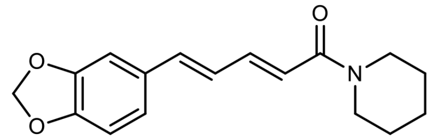

3

Supplementary Figure 1. Alkamides tentatively identified by LC-MS-QTOF from *P. nigrum* fruits. (**1**) piperettine, (**2**) trichostachine, **(3)** piperine, (**4**) piperolein A and **(5)** piperolein B.

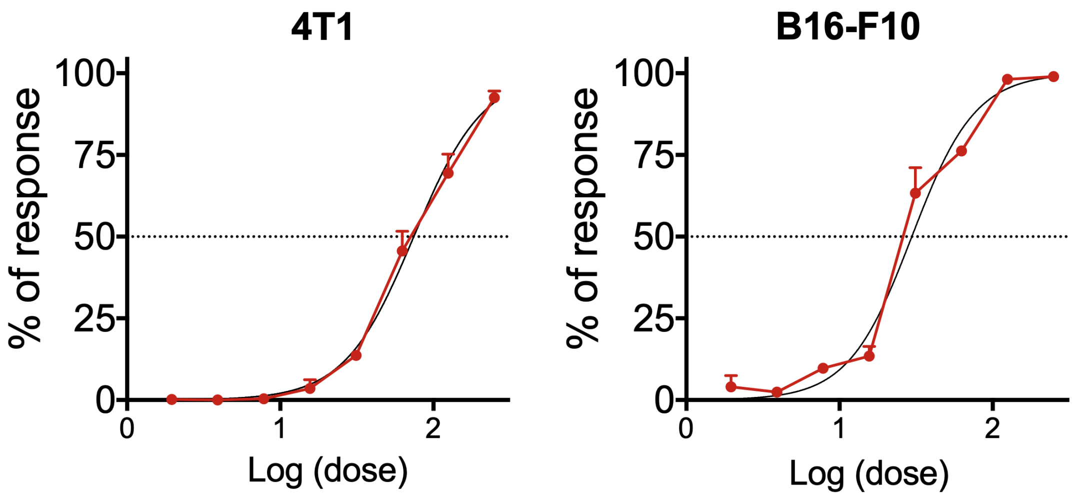

Supplementary Figure 2. Dose–response viability curve. Cells were seeded in 96-well plates and treated with different concentrations of *P. nigrum* extract for 48 h. Viability were determined by the MTT method described in Materials and Methods. The IC_{50} value was calculated using GraphPad Prism version 8.1.1 for Mac OS X statistics software (GraphPad Software, San Diego, CA). Black line: nonlinear regression curve fitting.

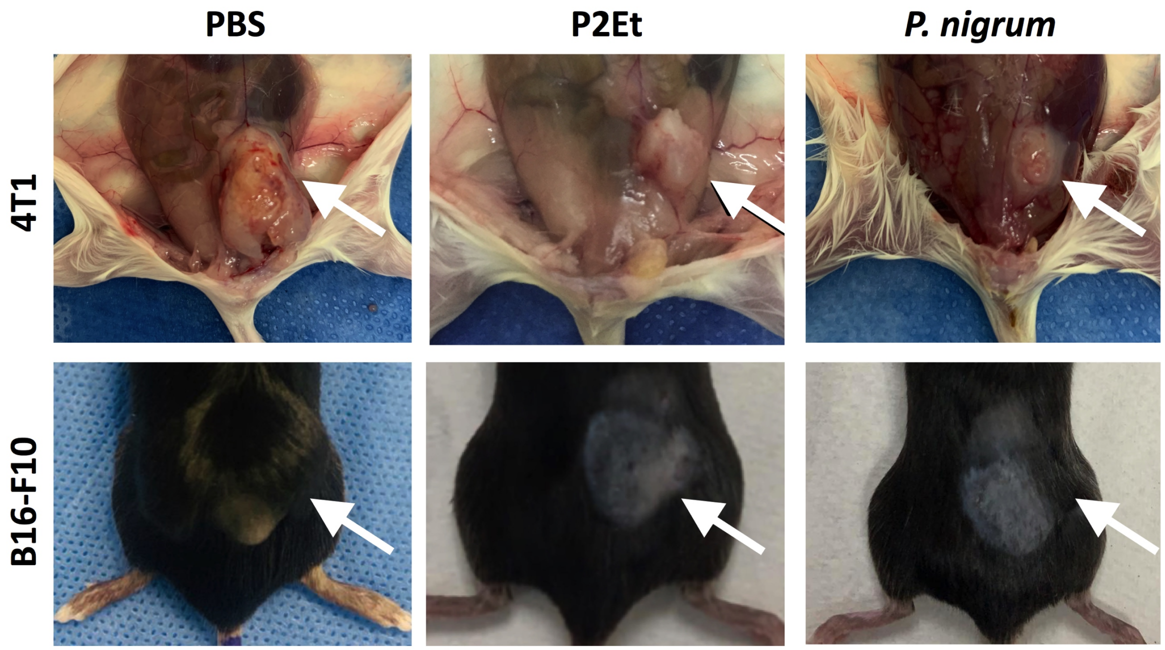

Supplementary Figure 3. Representative images. A. Representative images of tumor size in each treatment group of each tumor model.

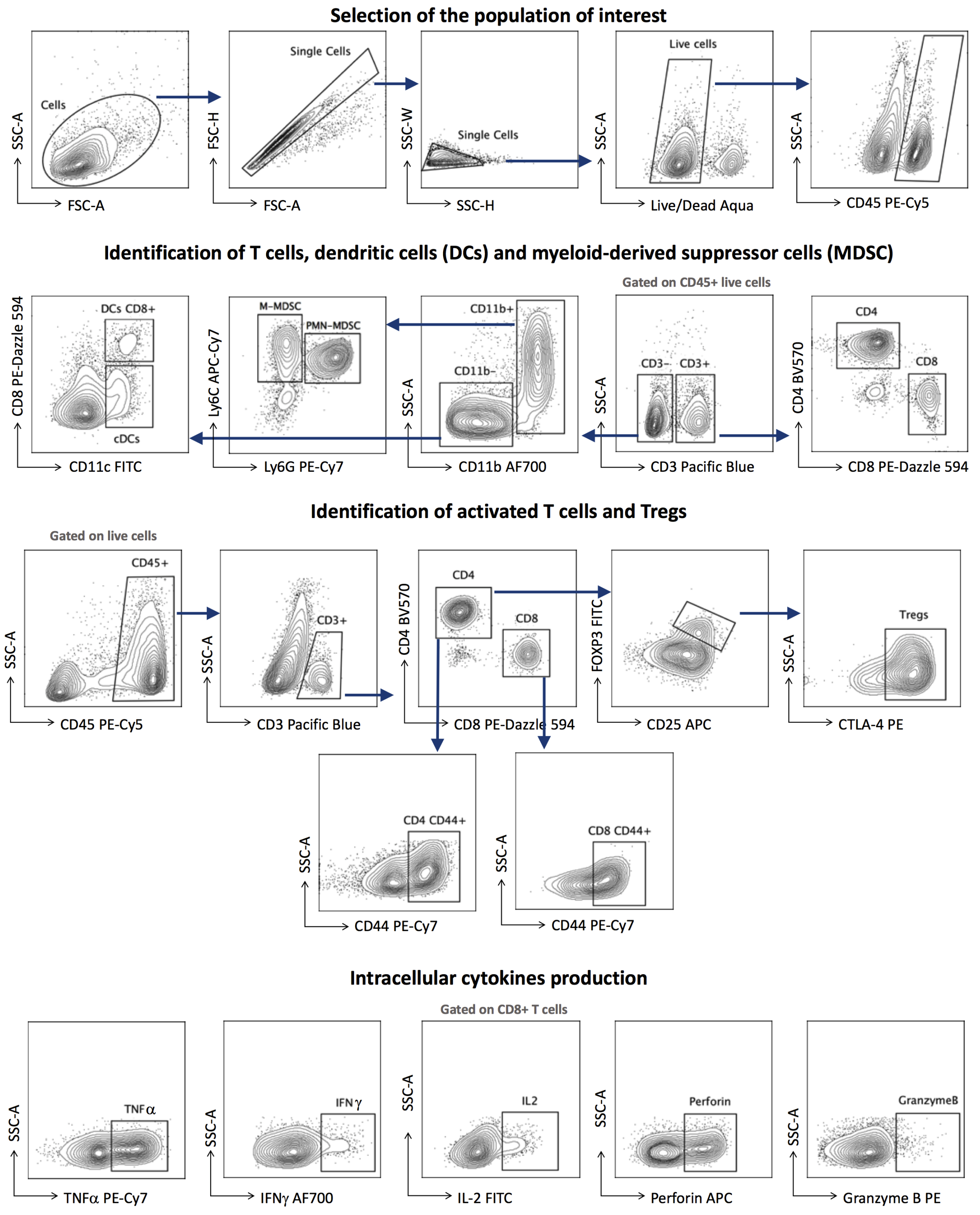

Supplementary Figure 4. Representative flow cytometry plots of each immune subpopulation evaluated and cytokine production.
